# A Noninvasive Aid for Office-Based Gynecologists for the Diagnosis of Common External Genital Disorders

**DOI:** 10.1155/2019/1830245

**Published:** 2019-10-16

**Authors:** Giorgia Giuffrida, Francesco Lacarrubba, Simona Boscaglia, Maria Rita Nasca, Giuseppe Micali

**Affiliations:** Dermatology Clinic, University of Catania, Catania, Italy

## Abstract

**Background:**

Gynecology and dermatology share the diagnosis and the management of some disorders of the female external genital area. In the last decade, clinical diagnosis in dermatology has dramatically improved, thanks to the introduction of dermatoscopy.

**Technique:**

Dermatoscopy is a noninvasive, rapid, and simple technique performed with an affordable handheld instrument called dermatoscope, endowed with a light source and a high-quality lens achieving 10 times magnification.

**Experience:**

The use of dermatoscopy for the diagnosis of some common external genital disorders, i.e., genital warts (GW), vestibular papillomatosis (VP), molluscum contagiosum (MC), angiokeratoma (AK), and pediculosis pubis (PP), is presented and discussed.

**Conclusion:**

The use of dermatoscopy in a gynecologic office may considerably help the specialist to enhance in selected cases the clinical diagnosis and to avoid unnecessary and cumbersome investigations which may be time and money consuming.

## 1. Introduction

Gynecology and dermatology share the diagnosis and the management of some disorders of the female genital area as it represents an anatomical region of common interest. In the last decade, clinical diagnosis in dermatology has dramatically improved, thanks to the introduction of dermatoscopy, a new noninvasive technique [1]. This article is aimed to provide an aid to gynecologists with some basic information on the use of dermatoscopy in selected external genital disorders in order to enhance the clinical diagnosis, thus sparing efforts and economic resources for nonessential investigations and treatments. Diagnostic clues for genital warts (GW), vestibular papillomatosis (VP), molluscum contagiosum (MC), angiokeratoma (AK), and pediculosis pubis (PP) are presented and discussed according to the recent findings in the literature and to personal experience.

## 2. Technique

**Figure 1:**
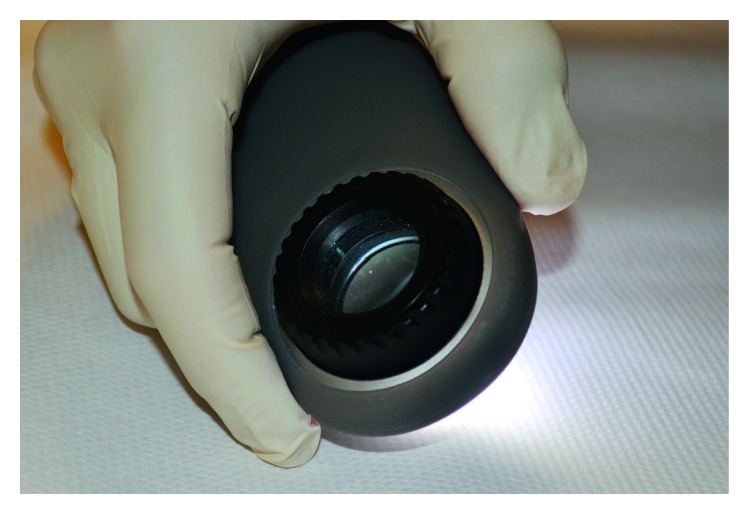
Common handheld dermatoscope.

Dermatoscopy is a noninvasive, rapid, and simple technique performed with an affordable (from ∼USD 600) handheld instrument called dermatoscope, endowed with a light source and a high-quality lens achieving 10 times magnification (Figure 1). It allows the detection of subsurface structures and the evaluation of diagnostic skin patterns not visible at naked eye observation [1]. A digital version connected to a personal computer (videodermatoscopy) provides higher magnification (up to 1000x) and direct visualization on a video terminal and immediate storage of images for successive comparisons, thus expediting the task of clinical and posttreatment follow-up. Both dermatoscopes and videodermatoscopes may be provided with polarized and nonpolarized light. Polarized devices should be preferred, especially in the genital area, as they do not require skin contact, thus avoiding transmission of infective disorders. Dermatoscopy has first been introduced for the differential diagnosis of pigmented lesions, but later, its use has been extended to encompass neoplastic, inflammatory, and infectious conditions [1].

## 3. Common External Genital Disorders

### 3.1. Genital Warts

**Figure 2:**
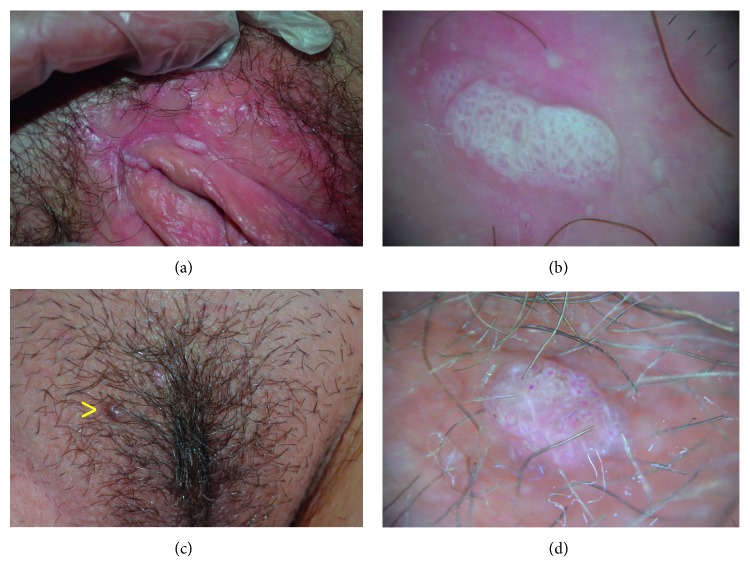
(a) Typical vulvar genital warts. (b) Dermatoscopy showing a white reticular network circumscribing areas centered by dotted vessels (“mosaic” pattern). (c) A nonspecific dome-shaped papule (arrow) in proximity of a genital wart of the vulva. (d) Dermatoscopy addressing to the diagnosis of genital wart by showing the characteristic “mosaic” pattern.

GW (also known as condylomata acuminata) are common communicable benign growths of the genital and perigenital areas, more often observed in young and sexually active individuals, caused by infection from human papilloma viruses (HPVs), most commonly HPV 6/11. They represent one of the most frequent sexually transmissible diseases. Clinically, GW appear as raised, variably sized and colored, soft, and fleshy papules, occurring as single elements or in clusters (Figure 2(a)), often showing elongated finger-like projections or a cauliflower-like aspect. The diagnosis is usually simple, however, early detection of initial minimal lesions or diagnosis of clinically misleading lesions may sometimes be challenging (Figure 2(c)), as GW may go unnoticed or mimic other dermatologic conditions, such as VP, MC, AK, lymphangiomas, and epidermoid cyst.

Dermatoscopy of GW varies depending on the clinical presentation. Papular lesions show the so-called “mosaic pattern,” consisting of a white reticular network circumscribing areas centered by dotted vessels (Figures 2(b) and 2(d)); finger-like and cauliflower-like lesions reveal multiple, irregular whitish projections that arise from a common base and comprise elongated and dilated vessels. Histopathologically these structures correlate with a variable degree of acanthosis and papillomatosis, along with the presence of elongated and tortuous vessels [2, 3].

### 3.2. Vestibular Papillomatosis

**Figure 3:**
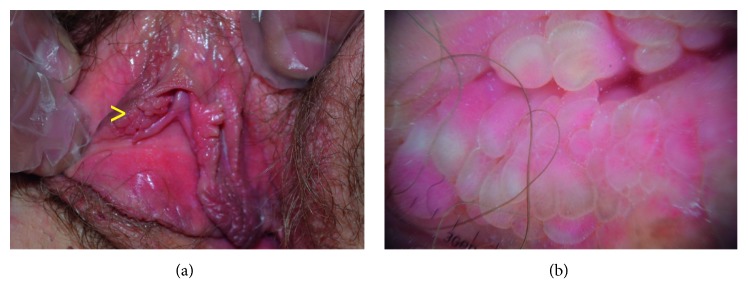
(a) Clinical presentation of vestibular papillomatosis. (b) Dermatoscopy showing regular and linear pinkish projections displaying a separated base.

VP (also known as hirsutoid vulvar papillomas, vulvar squamous papillomatosis, micropapillomatosis labialis, and squamous vestibular micropapilloma) is the female equivalent of pearly penile papules in men. It is a common and benign condition present in about 1% of women seeking gynecological advice. Clinically, VP appear as soft, flesh-colored, symmetric, and asymptomatic papules lining both sides of the vulva that generally remain unchanged with time (Figure 3(a)). The main differential diagnosis is represented by GW, but also by sebaceous glands, Bartolin's cysts, acrochordon, and MC. Moyal-Barracco et al. proposed five criteria to differentiate VP from GW: symmetric distribution, color similar to adjacent skin, separated base for any singular projection, soft consistency, and negative acetic acid test [4].

Dermatoscopy is a useful tool to differentiate these two conditions, as it allows optimal identification of some of these criteria. In VP, and not in GW, it clearly shows pinkish projections, regularly distributed and linearly arranged in a symmetric distribution, displaying a separated base; linear vessels may sometimes be observed (Figure 3(b)) [5]. Histological examination of VP displays multiple finger-like elongated structures, corresponding to the pinkish projections, with a moderately acanthotic epithelium overlying a central loose fibrovascular axis, resulting at dermatoscopy in the presence of linear vessels.

### 3.3. Molluscum Contagiosum

**Figure 4:**
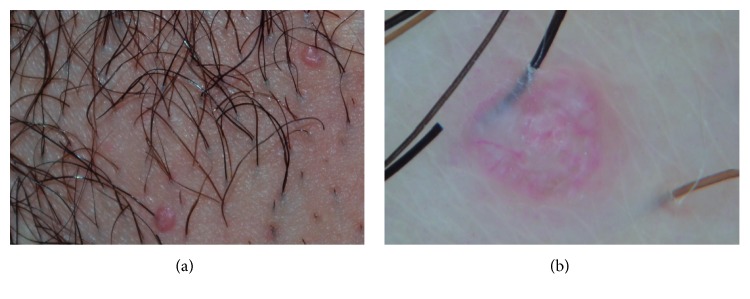
(a) Clinical presentation of 2 papules on the vulvar area resembling molluscum contagiosum. (b) Dermatoscopy showing a central yellowish-white, amorphous structure with a peripheral crown of vessels.

MC is a common skin infection caused by a Poxvirus. It mostly affects children but may also be observed on the genital areas or in its close proximity (lower abdomen or upper thigh), in sexually active individuals (15–29 years). MC appears as a small, pink to skin-colored, dome-shaped papule, with a central umbilication; the lesions are often multiple and grouped (Figure 4(a)). The diagnosis is usually clinic but in case of lesions showing unusual morphology, dermatoscopy may rule out GW, epidermal cyst, syringoma, closed comedo, and granuloma.

At dermatoscopy, MC shows a typical pattern consisting of a central yellowish-white, lobulated, amorphous structure with a peripheral crown of linear, fine, and sometimes blurred vessels that do not cross the center of the lesion (Figure 4(b)) [1, 6]. Histopathologically, the central polylobular structure correlates with the lobulated epidermal hyperplasia, whereas the vascular pattern corresponds to the dilated vessels in the dermis.

### 3.4. Angiokeratoma

**Figure 5:**
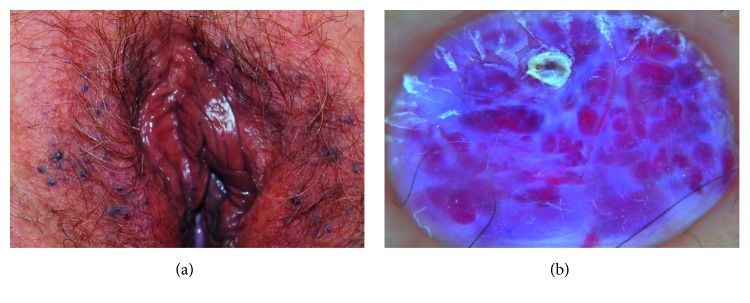
(a) Clinical presentation of angiokeratomas of the vulva. (b) Dermatoscopy of a papule showing red to dark lacunae and a whitish veil, surmounted by a microhemorrhagic crust.

AK of the vulva, unlike its male equivalent (scrotal AK), is considered a rare condition in women, more often reported between 20 and 40 years of age. It is a benign tumor characterized by numerous dilated dermal vessels with overlapping epidermal hyperplasia, which may occur as a single or multiple and disseminated lesions. AKs clinically present as keratotic papules of color ranging from red, reddish-blue to black (Figure 5(a)); they are usually asymptomatic, although pruritus, pain, burning, and bleeding are occasionally reported. Differential diagnosis includes GW, pyogenic granuloma, seborrheic keratosis, and melanoma.

Dermatoscopy can help to rule out these conditions showing the presence of typical red to dark lacunae, which are likely the result of dilated vessels with or without thrombosis, and of a whitish veil, that histologically corresponds to acanthosis or hyperkeratosis (Figure 5(b)). In addition, microhemorrhagic crusts and erythema may be observed [7].

### 3.5. Pediculosis Pubis

Pediculosis pubis (also known as pubic lice) is an ectoparasitosis caused by *Phthirus pubis* that generally occurs as a sexually transmitted infection. It peaks between 15 and 40 years, when sexual activity is higher, and has a male predilection probably because of a greater amount of body hairs that favours parasite transmission. The most frequently involved areas are the pubic and inguinal regions, but axillae and limbs may also be involved. Clinically, pruritus and scratching marks are major complaints. When present, maculae caeruleae, asymptomatic bluish-gray macules caused by crab bites, are a characteristic finding. Nits or lice grasped to the pubic hairs may be detected by simple naked eye inspection. Clinically, disorders that can mimic lice infestation are folliculitis, dermatophytosis, scabies, seborrheic scales, contact dermatitis, etc.

**Figure 6:**
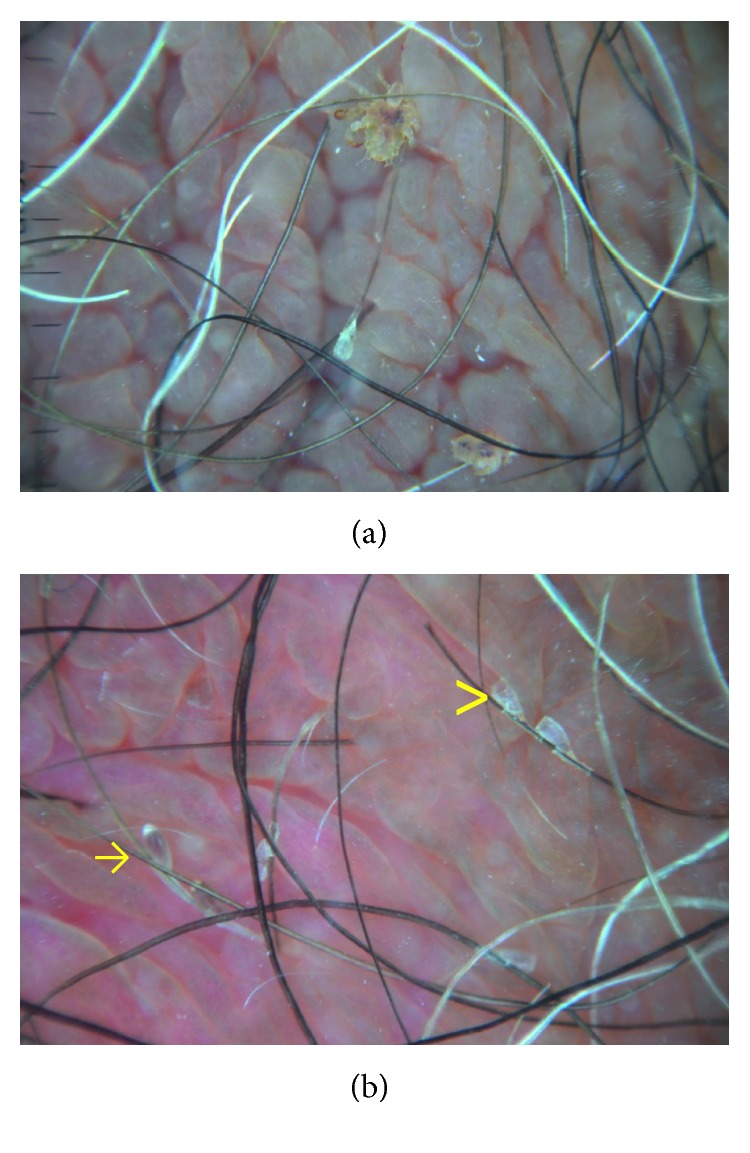
Dermatoscopy of pediculosis pubis. (a) Presence of 2 adult lice firmly attached to the hair shafts. (b) Multiple full (arrows) and empty (arrowheads) nits.

Dermatoscopy allows a clear and unequivocal visualization of both parasites (Figure 6(a)) and nits and permits to discriminate between full and empty nits: the former appear as ovoid, brown structures with a convex extremity, whereas the latter appear translucent and typically show a flat and fissured free ending corresponding to absence of parasites (Figure 6(b)) [1]. This information represents essential clues to a correct therapeutic approach.

## 4. Discussion

The diagnosis of common external disorders of the female genital area is generally made through an accurate visual inspection. The use of dermatoscopy, initially for the diagnosis of pigmented skin lesions including those appearing in the vulvar area [8], has considerably enhanced the simple clinical observation. With time, other fields of dermatology have adopted dermatoscopy as a tool capable to considerably increase physicians' clinical diagnostic skills, including skin infections and infestations (entodermoscopy), hair diseases (trichoscopy), nail disorders (onychoscopy), and inflammatory dermatoses (inflammoscopy) such as psoriasis and lichen planus [1]. Dermatoscopy has also been successfully used in some male genital disorders, such as GW, MC, pearly penile papules, Fordyce's spots, and median raphe cyst, demonstrating to heighten the clinical diagnosis [3, 9].

Given the high incidence of some external genital disorders as GW, which have a worldwide prevalence up to 44% in some studies, with an economic burden, in terms of diagnosis and treatment, of about 4 billion dollars a year only in the US [10], the possibility of extending the use of this new, noninvasive diagnostic tool in gynecology is desirable.

Dermatoscopy may therefore considerably help the office gynecologist to reach, in selected cases, the final diagnosis more easily so to avoid unnecessary and cumbersome investigations which may be time and money consuming. An extra advantage is that, in case of infective disorders, early diagnosis followed by a prompt eradication may considerably improve public health, timely suppressing potential sources of contamination/diffusion. Finally, a multidisciplinary approach with a strict collaboration between gynecologists and dermatologists should be encouraged for an optimal diagnostic approach to some genital disorders.
